# 

**Published:** 1895-02

**Authors:** 


					﻿Vol. XV.
FEBRUARY, 1895.
NO. 2.
THE
HOMEOPATHIC pHY^ClA]3,
A Monthly Journal of Homoeopathic Materia Medica
and Clinical Medicine.
“.He it a freeman whom truth maket free, and all are tlavet betide."
"Seek the Truth: come whence it may, cotl what it will."
KDJTKD AND PUBUBSKD BY
WALTER M. JAMES, M. E>.
PHILADELPHIA :
USTO, 1231 LOCIT8T STREET.
LONDON".
ALFRED HEATH & CO., Solb Abkntb ros Obbat Britain,
114 Ebury Street, Eaton Square.
29-Yearly Subtori ption, $2. SO, invariably in advance. Single numbere, 2S ett.
Entered al the Philadelphia Peet-Offlee an 8 wd-Claae Mall Matter.
				

